# A novel method for measuring absolute coronary blood flow and microvascular resistance in patients with ischaemic heart disease

**DOI:** 10.1093/cvr/cvaa220

**Published:** 2020-07-14

**Authors:** Paul D Morris, Rebecca Gosling, Iwona Zwierzak, Holli Evans, Louise Aubiniere-Robb, Krzysztof Czechowicz, Paul C Evans, D Rodney Hose, Patricia V Lawford, Andrew J Narracott, Julian P Gunn

**Affiliations:** 1 Mathematical Modelling in Medicine Group, Department of Infection, Immunity and Cardiovascular Disease, Medical School, University of Sheffield, Sheffield , UK; 2 Department of Cardiology, Sheffield Teaching Hospitals NHS Foundation Trust, Sheffield, UK; 3 Insigneo Institute for In Silico Medicine, University of Sheffield, Sheffield, UK; 4 The Bateson Centre, University of Sheffield, Sheffield, UK; 5 Department of Circulation and Medical Imaging, Norwegian University of Science and Technology (NTNU), Trondheim, Norway

**Keywords:** Coronary blood flow, Computational fluid dynamics, Coronary physiology, Coronary angiography, Coronary microvascular dysfunction

## Abstract

**Aims:**

Ischaemic heart disease is the reduction of myocardial blood flow, caused by epicardial and/or microvascular disease. Both are common and prognostically important conditions, with distinct guideline-indicated management. Fractional flow reserve (FFR) is the current gold-standard assessment of epicardial coronary disease but is only a surrogate of flow and only predicts percentage flow changes. It cannot assess absolute (volumetric) flow or microvascular disease. The aim of this study was to develop and validate a novel method that predicts absolute coronary blood flow and microvascular resistance (MVR) in the catheter laboratory.

**Methods and results:**

A computational fluid dynamics (CFD) model was used to predict absolute coronary flow (Q_CFD_) and coronary MVR using data from routine invasive angiography and pressure-wire assessment. Q_CFD_ was validated in an *in vitro* flow circuit which incorporated patient-specific, three-dimensional printed coronary arteries; and then *in vivo*, in patients with coronary disease. *In vitro*, Q_CFD_ agreed closely with the experimental flow over all flow rates [bias +2.08 mL/min; 95% confidence interval (error range) −4.7 to +8.8 mL/min; *R*^2^ = 0.999, *P* < 0.001; variability coefficient <1%]. *In vivo*, Q_CFD_ and MVR were successfully computed in all 40 patients under baseline and hyperaemic conditions, from which coronary flow reserve (CFR) was also calculated. Q_CFD_-derived CFR correlated closely with pressure-derived CFR (*R*^2^ = 0.92, *P* < 0.001). This novel method was significantly more accurate than Doppler-wire-derived flow both *in vitro* (±6.7 vs. ±34 mL/min) and *in vivo* (±0.9 vs. ±24.4 mmHg).

**Conclusions:**

Absolute coronary flow and MVR can be determined alongside FFR, in absolute units, during routine catheter laboratory assessment, without the need for additional catheters, wires or drug infusions. Using this novel method, epicardial and microvascular disease can be discriminated and quantified. This comprehensive coronary physiological assessment may enable a new level of patient stratification and management.

## 1. Introduction

Ischaemic heart disease is caused by restricted coronary blood flow. Thus, measurements of coronary blood flow would be helpful to guide clinical interventions in the catheter laboratory. Coronary flow however, is challenging to measure and there are no methods for measuring it in routine clinical use. Conversely, measurement of intracoronary pressure is simple, accurate, and reproducible. Consequently, cardiologists use translesional pressure measurements as a proxy for changes in blood flow. Examples include fractional flow reserve (FFR) and instantaneous wave-free ratio (iFR). These pressure-derived, surrogate flow indices are used widely to estimate the blood flow reduction due to epicardial coronary disease and guide the appropriateness of percutaneous coronary intervention (PCI). Use of FFR and iFR to guide PCI has become established in routine interventional practice and, compared with traditional angiography, has improved clinical outcomes.^1–3^

Such is the popularity and efficacy of these pressure-derived indices, that it is perhaps easy to overlook some limitations. First, FFR and iFR focus exclusively on the epicardial arteries and they cannot discriminate, or quantify microvascular disease. This is a significant limitation because coronary microvascular dysfunction (MVD) affects >50% of those assessed in the catheter laboratory, is prognostically important, is implicated in the 20% of patients with persistent angina after revascularization, affects women disproportionality, consumes excessive healthcare resources, and responds to European Society of Cardiology (ESC) guideline-indicated treatment.^4–9^ Yet, because it is overlooked by pressure-derived indices, MVD remains undiagnosed and untreated in many patients.^9^ Second, FFR and iFR predict a *percentage* flow restriction, but of an unknown value. They do not measure the actual (absolute) flow reduction in mL/min. Because relieving ischaemia is the main target for PCI,^10^^,^^11^ the ability to measure flow reduction in absolute terms may be beneficial. Unless absolute flow is measured, the true magnitude of the flow reduction cannot be known. Whilst there may be a broad correlation between FFR and absolute flow restriction, knowing whether an FFR of 0.80 represents a 20 or 120 mL/min reduction in coronary flow can only add value to the coronary physiological assessment.

If both pressure *and* flow could be measured reliably and simply, a number of additional physiological parameters could be calculated using basic haemodynamic laws. These include microvascular resistance (MVR), stenosis resistance (SR), and coronary flow reserve (CFR), all of which help to discriminate and independently assess microvascular and epicardial coronary disease, thus providing a comprehensive physiological assessment of the entire coronary circulation.

To enable this ‘next-level’ of coronary physiological assessment, there is therefore, a need for a method that measures absolute coronary blood flow, in combination with intracoronary pressure, practical for routine use in the cardiac catheter laboratory. The aim of this study was to develop and validate a novel computational fluid dynamics (CFD) based method which, with a standard pressure wire, could assess absolute coronary blood flow, and all relevant coronary physiological indices, including MVR.

## 2. Methods

This research was performed at the University of Sheffield and Sheffield Teaching Hospitals NHS Foundation Trust UK, conformed to the principles of the Declaration of Helsinki and was approved by the NHS Health Research Authority, Regional Ethics Committee. Participating patients provided informed consent.

### 2.1 The computational model

**Figure 1:**
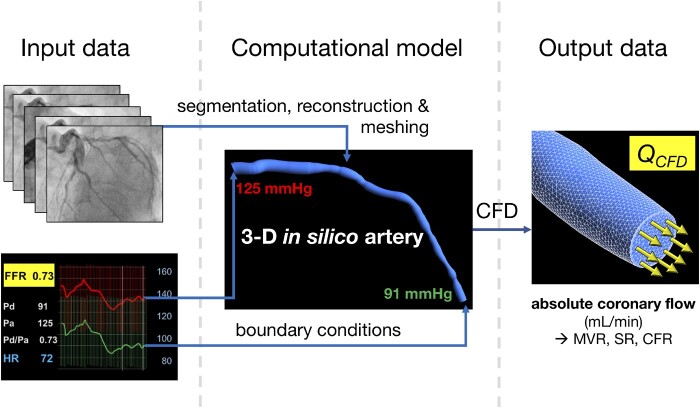
The computational method for computing absolute coronary blood flow. Coronary angiographic images are used to reconstruct the coronary anatomy. Pressure data are used to tune boundary conditions. CFD simulation computes the volumetric flow rate (Q_CFD_), which enables coronary microvascular resistance (MVR), stenosis resistance (SR), and coronary flow reserve (CFR) to be calculated automatically.

Model inputs were standard coronary angiographic (digital imaging and communications in medicine, DICOM) images and pressure data. The principal model output was absolute coronary flow (Q_CFD_) in mL/min. Three-dimensional coronary anatomy was reconstructed within the virtuQ software from two, two-dimensional angiographic projections, acquired ≥30° apart during end-diastole, producing an axisymmetric three-dimensional (3D) model.^12^^,^^13^ Volume mesh was constructed with 1.2–1.5 M elements. Pressure boundary conditions were applied at the inlet and outlet, informed by the invasively measured values.^14^ CFD simulation was performed (ANSYS, PA, USA) on a Dell Precision T5600 computer (Intel Xeon E5 2650, 2 GHz processor, 32GB RAM) to a residual target of 10^−6^.^12^ The arterial wall was considered rigid. The Q_CFD_ method is outlined in *Figure 1*. Using the hydraulic equivalent of Ohm’s law, Q_CFD_ and pressure data were used to calculate coronary MVR, SR, and CFR under baseline (BL) and hyperaemic (Hyp) conditions as follows:

$$\begin{array}{l} MVR\;=\;\frac{P_{d}}{Q_{CFD}}\\ SR\;=\;\frac{P_{a}-P_{d}}{Q_{CFD}}\\ CFR\;=\;\frac{Q_{CFD}^{Hyp}}{Q_{CFD}^{BL}} \end{array}$$

### 2.2 *In vitro* assessment

**Figure 2:**
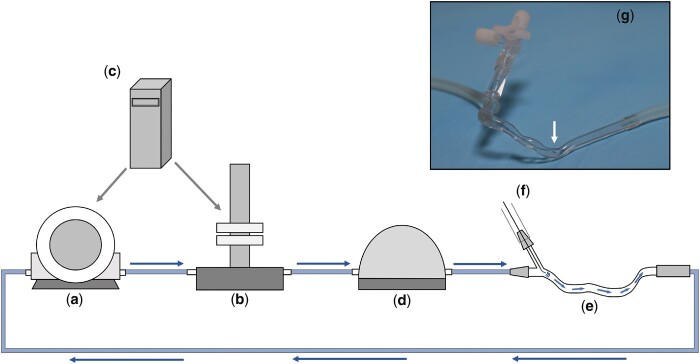
The in vitro test-rig used for validating the method for determining absolute flow. A gear pump (a) (TA Instruments, USA) delivered steady flow through the circuit. In the pulsatile experiments, a pulsatile manifold (Bose Corp, USA) was used to deliver pulsatile flow. Both devices were controlled by WinTest^®^ software (Bose) (c). A compliance chamber (d) was used in tandem with the pump and manifold to remove high frequency signal artefact. The blood analogue fluid (40/60 glycerol/water, viscosity 0.0035 Pa⋅s, 1082 kg⋅m^−3^ at room temperature)^15^ passed through the 3D printed artery (e) reconstructed from patient data. Clinical haemostatic valves were used to instrument the system with pressure and flow transducers through f. The photographs demonstrate a 3D printed artery within the circuit and the pressure wire tip can be seen on the zoomed image (g). The flow rate was regularly calibrated by measuring the volume of fluid draining into the reservoir chamber (not seen in this idealized diagram).

Q_CFD_ accuracy was validated in an *in vitro* flow circuit outlined in *Figure 2*. To provide realistic experimental conditions, patient-specific coronary arteries were 3D printed. Cases included left anterior descending artery (LAD), right coronary artery (RCA), and left circumflex artery (LCX). Percentage diameter stenosis ranged from 46% to 72% and lengths ranged from 68 mm to 84 mm. Case-specific details of the individual models and of the 3D printing protocol can be found in the Supplementary material online. Flow rates were varied from 50 to 180 mL/min in 10 mL/min increments. Assuming a baseline flow of 60 mL/min and a CFR of up to three (in the context of flow-limiting lesions), this reflects a broad physiological range from baseline through hyperaemic conditions.

Proximal pressure (P_a_) was measured using a TruWave Pressure Transducer (Edwards Lifesciences Corp, USA) and distal pressure (P_d_) with a Volcano Primewire (Philips Volcano, Philips Healthcare, Best, Netherlands). Experimental flow rate (Q_Exp_) was repeatedly calibrated (prior to every analysis) by measuring the fluid volume draining into a flask in one minute. Coronary models were run at all 14 flow rates. Each was repeated three times, with a mean result recorded. Four-hundred and twenty analyses were performed in total. The pressure gradient (P_a_–P_d_) was applied as a pressure boundary condition at the inlet with zero pressure at the outlet. For Doppler analysis, an ultrafine nylon powder (Orgasol^®^ Powders, Arkema Group, Colombes, France) was added to the blood-analogue fluid to mimic the ultrasonic back-scatter properties of erythrocytes.^16^ Doppler flow velocity was measured with a Philips Volcano Doppler FloWire^®^ (Philips Volcano, CA, USA). The Doppler wire was positioned and manipulated until the optimal (most dense) Doppler signal was recorded. Coronary flow derived from Doppler ultrasound measurements (Q_Dop_) was calculated from Doppler flow average peak velocity (APV), assuming a parabolic laminar flow profile (APV = 2 × mean velocity), by considering the luminal cross-sectional area (*V* = *Q*/*A*), where *A* was known precisely from the print files.

The primary outcome measure was the accuracy of computed flow rate, Q_CFD_, compared with the calibrated Q_Exp_. Physiological flows are typically laminar [Reynolds (Re) number <500] but the experimental protocol had potential to induce supra-physiological flow rates (180 mL/min through severe stenosis).^17^ We therefore also report accuracy for the subset of cases where Reynolds number (Re) is less than 500 ($\text{Re}=\frac{\rho \text{VD}}{\mu }$ where *V* is the average velocity over the circular cross-section at the location with minimum diameter (stenosis), *D* the diameter at this location and ρ and μ the density and viscosity, respectively).

To investigate whether there was any additional value in terms of increased Q_CFD_ accuracy in simulating pulsatile flow we also ran all the models at all flow rates under both steady and pulsatile flow profiles and simulated likewise in the computational model. Mean P_a_ and P_d_ were applied for steady analysis, and transient P_a_ and P_d_ measurements (with transient analysis) for pulsatile. Details regarding how flow pulsatility was imposed in the flow circuit can be found in the Supplementary material online.

### 2.3 First-in-man *in vivo* assessment

Angiographic and invasive pressure data were collected from a previously unstudied cohort of 40 patients with stable coronary artery disease. Patients with history of coronary artery bypass surgery were excluded. Q_CFD_ was computed using the computational model as described above under baseline and hyperaemic conditions with time averaged P_a_ and P_d_ applied as the inlet and outlet boundary conditions, respectively measured simultaneously from the pressure wire and guide catheter. MVR, SR, and CFR were calculated using the equations above. An independent operator repeated 24 Q_CFD_, MVR, and Q_CFD_-derived CFR analyses to derive interobserver variability. A subset of 20 patients also underwent Doppler flow wire (FloWire^®^, Philips Volcano, NL) assessment, from which coronary flow (Q_Dop_) and CFR ($Q_{\text{Dop}}^{\text{Hyp}}/Q_{\text{Dop}}^{\text{BL}}$) were derived. Measurements were repeated three times and the mean value was recorded. Pressure-derived CFR (CFR_P-D_) was also calculated according to:

$${\text{CFR}}_{P-D}=\sqrt{P_{a}–P_{d}\;\text{hyperaemic}\;}/\sqrt{P_{a}–P_{d}\;\text{baseline}}.$$

CFR_P-D_ is known to correlate closely with CFR.^18^ CFR_P-D_ was therefore compared with CFR derived from the novel Q_CFD_ method (CFR_QCFD_) and that derived from Doppler (CFR_Dop_). We also compared the pressure drop computed by the computational model with flow applied as the inlet boundary condition using both Q_CFD_ and Q_Dop_.

### 2.4 Statistical analysis

Unless stated otherwise, mean delta and standard deviation (SD) of the mean are presented. Agreement was assessed using Bland–Altman plots. Bland–Altman limits of agreement (±1.96 SD), which comprise 95% of all results, were used as the error range.^19^ Reproducibility was assessed by calculating the coefficient of variation (CoV) as the ratio of the standard deviation and mean values of repeated samples. Pearson coefficient (*r*) was used to calculate linear correlation and *R*^2^. Seventeen or more paired samples were required to detect *r* ≥ 0.70 at 0.05 significance and 0.90 power. Analysis was performed using SPSS (IBM Corp, USA).

## 3. Results

### 3.1 Accuracy of steady CFD analysis

In all cases, over all flow rates, the difference between steady and pulsatile flow was negligible (bias −0.2 mmHg SD 0.9 mmHg equating to <1 mL/min difference). Given that steady CFD analysis, based on time-averaged pressure boundary conditions is considerably quicker and simpler to compute, we elected to use this model for the (*in vivo*) analysis. Further details of this analysis can be found in the Supplementary material online.

### 3.2 *In vitro* assessment: Q_CFD_ predicts absolute flow

**Figure 3:**
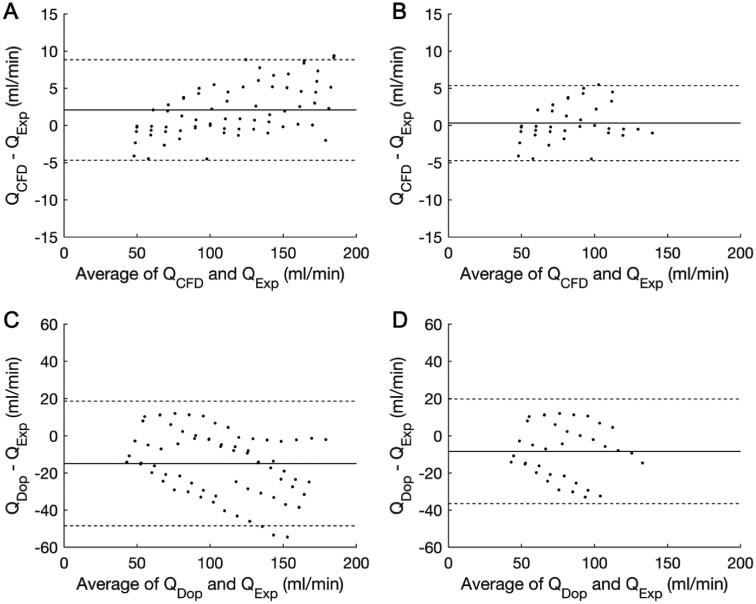
Bland–Altman plots demonstrating the accuracy of Q_CFD_ and Q_Dop_. (*A*) The accuracy of the novel Q_CFD_ method over all flow rates [bias +2.08 mL/min; limits of agreement (±1.96 SD) ±6.75 mL/min]. (*B*) The accuracy of Q_CFD_ for cases with Re ≤ 500 (bias +0.31 mL/min; limits of agreement ±5.0 mL/min). (*C*) The accuracy of the Doppler method (Q_Dop_) over all flow rates (bias −14.9 mL/min; limits of agreement ±33.5 mL/min). (*D*) The accuracy of the Doppler method for cases with Re ≤ 500 (bias −8.34 mL/min; limits of agreement ±28.1 mL/min). The solid line indicates the bias (mean delta) and the broken lines indicate the limits of agreement (±1.96 SD). Both methods were plotted against the gold-standard of the calibrated experimental flow rate (Q_exp_). Note the difference in *Y*-axis scale between the two methods. Each dot represents the average of three recordings, i.e. 70 data points and 210 samples.

**Figure 4:**
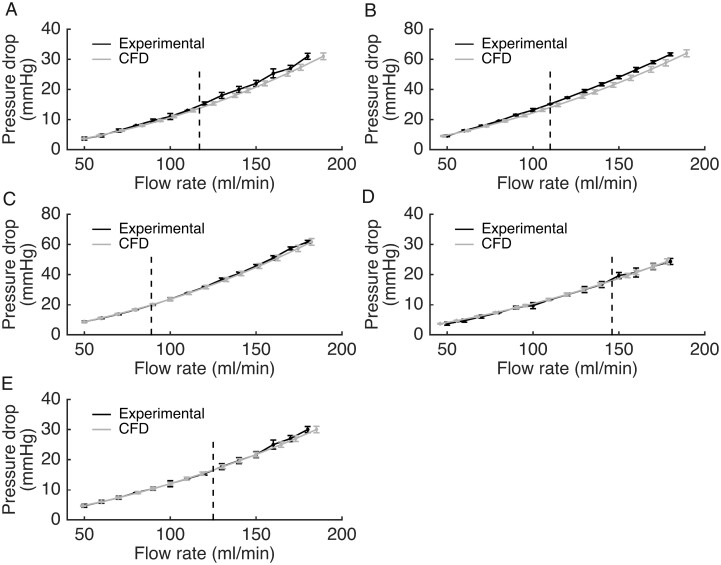
Pressure gradient vs. flow during *in vitro* testing for each of the five models over all flow rates. There was close agreement between the experimental (gold-standard) flow (Q_exp_) indicated by the black line and the flow rate computed by the novel method (Q_CFD_) indicated by the grey line (*R*^2^ 0.999, *P* < 0.001, by Pearson’s correlation coefficient). The vertical dashed line represents the transition between physiological (Re < 500) and supra-physiological (Re ≥ 500) flow rates. For Q_exp_, error bars represent the maximum and minimum values obtained from three measurements. Because CFD results are inherently reproducible given identical setup parameters, error bars for the Q_CFD_ model were calculated from simulation data representing the influence of small errors in viscosity and density of the experimental blood analogue. Each data point represents the mean of three repeated measurements, i.e. 42 samples per model and 210 all together.

Analysis in the 3D printed coronary artery geometries revealed close agreement and correlation between Q_CFD_ and Q_Exp_ (mean delta +2.08 mL/min, SD 3.45 mL/min, limits of agreement −4.7 to +8.8 mL/min, *R*^2^ 0.999; *P* < 0.001) (*Figures 3 and**4*). Q_CFD_ results were reproducible over three repeated measurements and CFD analyses (CoV <1.0%). When cases with Reynolds numbers >500 were excluded, accuracy improved (mean delta +0.31 mL/min, SD 2.58 mL/min, limits of agreement −4.7 to +5.3 mL/min) (*Figure 3*). Results for the individual models are provided in the Supplementary material online. The mean CFD processing time for all analyses was 189 s which is tractable for on table clinical decision making.

### 3.3 Accuracy of Doppler flow

The coefficient of variability for Doppler flow (Q_Dop_) was 6.4% when the wire was positioned at the inlet, and 17.4% distal to the stenosis. Accordingly, only inlet measurements were considered further. Despite a strong correlation (*R*^2^ 0.98; *P* < 0.001), Q_Dop_ underestimated Q_Exp_ (mean delta −14.9 mL/min) and limits of agreement were wider than Q_CFD_ (−48.4 to +18.6 mL/min) (*Figure 3*). Accuracy of Q_Dop_ was only improved slightly when cases with Re > 500 were excluded (mean delta −8.34 mL/min, limits of agreement −36.4 to +19.8 mL/min). Thus, we conclude that in the in vitro assessment, the novel Q_CFD_ method demonstrated close agreement and correlation with the actual flow rate with high reproducibility. Q_CFD_ was considerably more accurate and reproducible than the Doppler wire method.

### 3.3 First-in-man *in vivo* assessment of Q_CFD_ and MVR

Forty patients were studied during invasive coronary angiography. Mean age was 65 (±6) years and 86% were male. Medical history included hypertension in 63%, Type 2 diabetes mellitus in 32%, and treated dyslipidaemia in 65%. In total, 13% were current smokers and 5% had experienced prior myocardial infarction. The arteries studied were 29 LAD (72.5%), 5 LCX (12.5%), 5 RCA (12.5%), and 1 left main stem (2.5%). Mean FFR was 0.78 (±0.12). Q_CFD_ was successfully computed in all cases under baseline and hyperaemic conditions. The mean baseline Q_CFD_ was 62.0 (±28) mL/min and mean hyperaemic Q_CFD_ was 92.4 (±46) mL/min. Q_CFD_ was used to additionally calculate coronary MVR, SR and CFR. Between baseline and hyperaemia, there was a 46% reduction in coronary MVR (1.62 ± 0.88 to 0.88 ± 0.45 mmHg⋅s⋅mL^−1^, *P* < 0.001) and a 21% rise in SR (0.19 ± 0.12 to 0.23 ± 0.14 mmHg⋅s⋅mL^−1^, *P* < 0.01). Mean CFR_QCFD_ was 1.56 (±0.44). Interobserver variability for Q_CFD_, MVR, and Q_CFD_-derived CFR was 10%, 11%, and 6%, respectively.

### 3.4. Accuracy in predicting pressure-derived CFR and reproducing the measured pressure gradient

**Figure 5:**
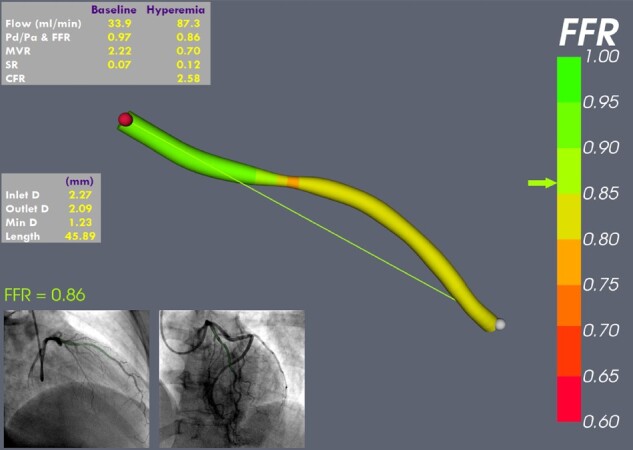
Example result from the virtuQ software graphical user interface. Absolute flow (mL/min), resting Pd/Pa, FFR, microvascular resistance (MVR), stenosis resistance (SR), and coronary flow reserve (CFR) are reported alongside the angiogram images (for reference), interactive 3D reconstructed artery (physiologically colour mapped) and a 3D vessel-sizing application to facilitate potential stent choice. The operator can select any two points within the vessel and the results update live. In this case, the FFR is negative, but flow, according to CFR, is borderline, likely due to the increased MVR.

CFR derived from the novel method (CFR_QCFD_) correlated closely with pressure-derived CFR (CFR_P-D_) (*R*^2^ 0.92, *P* < 0.001). CFR_P-D_ systematically underestimated CFR_QCFD_ (mean delta −0.16 ± 0.17). The measured and computed physiological parameters of all 40 patients are reported in *Table 1*. Doppler assessment was attempted in a subset of 20 patients but signal quality was inadequate for CFR estimation in two cases (10%). In the remaining 18, the correlation between CFR derived from Q_Dop_, (CFR_Dop_), and CFR_P-D_ were weak (*R*^2^ 0.32, *P* = 0.1). Similar to CFR_QCFD_, CFR_Dop_ also overestimated CFR_P-D_ (mean delta −0.35 ± 0.46). When the computational model was reversed to apply flow at the inlet, application of Q_CFD_ accurately predicted the invasively measured pressure gradients (bias −0.29, SD 0.46 mmHg, limits of agreement −1.19 to +0.61 mmHg), whereas Q_Dop_ consistently underestimated the invasively measured pressure gradient (bias −8.93, SD 12.46 mmHg limits of agreement −33.35 to +15.49 mmHg). Unlike the *in vitro* experiments, this does not provide a rigorous validation of flow results, but it does suggest that the Q_CFD_ method was reasonably accurate relative to Doppler. *Figure 5* demonstrates a screenshot of a result within the virtuQ software environment.

**Table 1:** Measured and computed physiological parameters for all 40 cases

Case		Baseline							Hyperaemic							BL-Hyp % delta			CFR		
No	Artery	Pa	Pd	dP	Pd/Pa	Q_CFD	MVR	SR	Pa	Pd	dP	FFR	Q_CFD	MVR	SR	MVR	SR	Q	Dop	Q_CFD	P-D
1	LAD	88.0	79.4	8.7	0.90	22.7	3.49	0.38	78.0	61.0	17.0	0.78	35.8	1.70	0.47	−51	24	58	1.8	1.58	1.40
2	LAD	92.7	85.6	7.1	0.92	67.7	1.26	0.10	75.7	61.5	14.2	0.81	109.2	0.56	0.13	−56	30	61	1.3	1.61	1.41
3	RCA	106.3	99.6	6.7	0.94	89.8	1.11	0.08	83.5	71.3	12.2	0.85	165.3	0.43	0.07	−61	−13	84	2.1	1.84	1.35
4	LAD	119.9	114.7	5.1	0.96	72.9	1.57	0.07	114.9	91.7	23.1	0.80	193.0	0.48	0.12	−69	71	165	2.4	2.65	2.13
5	RCA	89.1	66.4	22.7	0.74	93.2	0.71	0.24	88.3	53.8	34.5	0.61	121.9	0.44	0.28	−38	17	31	1.5	1.31	1.23
6	LCX	74.3	69.1	5.2	0.93	36.6	1.89	0.14	77.4	60.0	17.5	0.77	88.7	0.68	0.20	−64	43	142	1.1	2.42	1.83
7	LAD	76.7	51.8	24.9	0.68	89.0	0.58	0.28	65.2	33.1	32.2	0.51	105.4	0.31	0.31	−47	11	19	1.1	1.19	1.14
8	LAD	99.0	80.9	18.1	0.82	75.4	1.07	0.24	104.6	73.6	31.0	0.70	108.9	0.68	0.28	−36	17	44	1.5	1.44	1.31
9	LAD	96.4	87.6	8.8	0.91	36.6	2.40	0.24	71.4	62.7	8.7	0.88	36.3	1.73	0.24	−28	0	−1	2.40	0.99	0.99
10	LAD	117.3	112.9	4.4	0.96	23.5	4.80	0.19	102.3	89.0	13.3	0.87	52.8	1.69	0.25	−65	32	125	2.07	2.25	1.74
11	RCA	90.0	88.0	1.9	0.98	41.3	2.13	0.05	75.0	67.6	7.4	0.90	102.5	0.66	0.07	−69	40	148	1.90	2.48	1.97
12	RCA	112.1	59.4	52.7	0.53	115.1	0.52	0.46	118.8	62.2	56.5	0.52	119.7	0.52	0.47	0	2	4	1.18	1.04	1.04
13	LAD	112.6	106.9	5.7	0.95	58.0	1.84	0.10	110.4	103.4	7.0	0.94	66.4	1.56	0.11	−15	10	14	1.38	1.14	1.11
14	RCA	106.5	105.8	0.7	0.99	29.7	3.56	0.02	103.7	102.0	1.7	0.98	60.1	1.70	0.03	−52	50	102	2.42	2.02	1.56
15	LAD	99.6	80.3	19.3	0.81	46.4	1.73	0.42	97.0	69.9	27.1	0.72	58.2	1.20	0.47	−31	12	25	1.61	1.25	1.18
16	LAD	109.9	92.9	17	0.85	86.8	1.07	0.20	103.1	86.1	17.0	0.84	86.7	0.99	0.20	−7	0	0	1.67	1.00	1.00
17	LAD	110.3	98.4	11.9	0.89	55.6	1.68	0.21	106.4	85.0	21.4	0.80	79.1	1.01	0.27	−40	29	42	1.2	1.42	1.34
18	LCX	108.1	94.5	13.6	0.87	85.0	1.05	0.16	113.7	88.5	25.2	0.78	121.2	0.69	0.21	−34	31	43	2	1.43	1.36
19	LAD	96.4	87.6	8.8	0.91	84.1	0.98	0.10	71.5	62.4	9.1	0.87	85.2	0.67	0.11	−32	10	1	F	1.01	1.02
20	LAD	106.3	99.6	6.7	0.94	67.6	1.40	0.10	81.8	63.6	18.2	0.78	123.5	0.47	0.15	−66	50	83	F	1.83	1.65
21	LAD	88.6	79.5	9.1	0.90	27.1	2.75	0.34	80.9	58.9	22.0	0.73	47.7	1.13	0.46	−59	35	76		1.76	1.55
22	LAD	74.4	49.5	24.9	0.67	56.2	0.83	0.44	65.0	32.9	32.1	0.51	65.8	0.43	0.49	−48	11	17		1.17	1.14
23	LCX	73.6	68.3	5.3	0.93	38.7	1.64	0.14	79.1	60.8	18.3	0.77	81.6	0.68	0.22	−59	57	111		2.11	1.86
24	LAD	96.3	87.5	8.8	0.91	41.0	2.01	0.21	71.5	62.4	9.1	0.87	41.1	1.40	0.22	−30	5	0		1.00	1.02
25	LAD	99.0	80.9	18.1	0.82	57.4	1.32	0.32	104.4	73.5	30.9	0.70	79.8	0.86	0.39	−35	22	39		1.39	1.31
26	LAD	119.0	112.1	6.9	0.94	42.0	2.55	0.16	103.4	88.5	14.9	0.86	62.6	1.33	0.24	−48	50	49		1.49	1.47
27	LAD	112.3	106.7	5.6	0.95	61.5	1.65	0.09	122.0	113.1	8.9	0.93	82.2	1.32	0.11	−20	22	34		1.34	1.26
28	LAD	99.6	80.3	19.3	0.81	47.3	1.59	0.41	95.5	68.7	26.8	0.72	58.8	1.09	0.46	−31	12	24		1.24	1.18
29	LMS	78.8	64.7	14.1	0.82	169.4	0.35	0.08	75.3	47.4	27.9	0.63	257.4	0.16	0.11	−54	38	52		1.52	1.41
30	LAD	87.0	82.1	4.9	0.94	43.4	1.78	0.11	84.1	70.9	13.2	0.84	79.5	0.83	0.17	−53	55	83		1.83	1.64
31	LAD	81.5	73.0	8.5	0.90	44.1	1.54	0.19	78.3	58.2	20.1	0.74	78.2	0.68	0.26	−56	37	77		1.77	1.54
32	LAD	101.6	84.4	17.2	0.83	38.2	2.08	0.45	82.5	48.2	34.3	0.58	59.7	0.72	0.57	−65	27	56		1.56	1.41
33	LAD	93.5	86.3	7.2	0.92	79.4	0.97	0.12	87.0	62.0	25.0	0.71	148.7	0.38	0.17	−61	42	87		1.87	1.86
34	LAD	109.0	102.0	7.0	0.94	52.4	1.85	0.13	118.8	110.2	8.6	0.93	59.0	1.78	0.15	−4	15	13		1.13	1.11
35	LCX	130.4	119.4	11.0	0.92	79.6	1.44	0.14	118.0	101.0	17.0	0.86	105.2	0.91	0.16	−37	14	32		1.32	1.24
36	LCX	94.2	84.9	9.3	0.90	83.4	0.96	0.11	96.7	67.6	29.1	0.70	173.8	0.36	0.17	−63	55	108		2.08	1.77
37	LAD	99.7	90.5	9.2	0.91	75.1	1.14	0.12	98.2	84.8	13.4	0.86	92.5	0.86	0.14	−25	17	23		1.23	1.21
38	LAD	88.0	82.6	5.4	0.94	66.8	1.16	0.08	78.7	58.6	20.1	0.74	138.1	0.39	0.15	−66	88	107		2.07	1.93
39	LAD	67.1	59.8	7.3	0.89	50.4	1.09	0.14	69.8	59.4	10.4	0.85	63.7	0.85	0.16	−22	14	26		1.26	1.19
40	LAD	69.8	64.4	5.4	0.92	49.7	1.20	0.11	66.0	56.1	9.9	0.85	73.9	0.69	0.13	−43	18	49		1.49	1.35

BL, baseline; CFR, coronary flow reserve; Dop, Doppler; F, failed; FFR, fractional flow reserve; Hyp, hyperaemia; LAD, left anterior descending; LCX, left circumflex; LMS, left main stem; MVR, microvascular resistance; Pa, proximal pressure; Pd, distal pressure; P-D, pressure derived; Q_CFD, coronary flow computed by the novel method; RCA, right coronary artery; SR, stenosis resistance.

## 4. Discussion

In this study, we have demonstrated that absolute coronary blood flow can be determined from data generated during standard angiography and pressure wire assessment. In addition to absolute coronary flow, FFR, MVR, SR, and CFR can be determined simultaneously, providing a comprehensive physiological assessment of the key physiological parameters which characterize the entire coronary circulation. Uniquely, the method does not require any dedicated hardware, infusions or interventional effort. The novel method was more accurate and reproducible than the Doppler wire technique.

Indices of translesional pressure ratio like FFR and iFR are themselves methods for deriving flow from pressure and are superior to angiography in determining physiological lesion significance. However, flow is not measured, but inferred, based upon a number of assumptions. These indices reflect percentage changes in flow, of an unknown value, relative to a hypothetical norm. We propose there is value in understanding flow and flow reduction in absolute terms. An FFR of 0.75 indicates a 25% reduction of flow in that artery, compared with the undiseased state. Precisely how much blood flow this is cannot be known. This may be an important limitation, because an FFR of 0.75 in a diagonal branch may represent just a few mL/min of flow reduction, whereas the same FFR in a proximal LAD may indicate well over 100 mL/min flow reduction. Similarly, an FFR 0.78 in the diagonal branch might seem to mandate PCI, whereas an FFR of 0.82 in a proximal LAD would not, even if, in absolute terms, the LAD lesion is associated with a far greater reduction in absolute myocardial blood flow. The value of FFR is that it has allowed interventionists to begin to quantify blood flow reduction in the catheter laboratory, but the ability to accurately quantify coronary blood flow changes in absolute terms may enable a more refined and patient-specific approach to coronary physiological assessment and treatment decisions. Without any additional equipment than it takes to measure FFR, our novel computational method additionally reports (i) the flow reduction in absolute terms, (ii) the MVR, (iii) the SR, and (iv) the CFR. Thus, the new method does not compete with traditional parameters like FFR or iFR, but instead complements and augments them, providing a new level of coronary physiological information. Our method took between seven and eight minutes to complete using our software; four to five min to reconstruct the arterial geometry and three to compute the physiology. Speed of computation was not the focus of this study, rather accuracy of the novel method. We anticipate results can be achieved in less than 5 min with development of the user interface and accelerated CFD code.

An important advantage of the novel method is that it provides information regarding microvascular disease. The importance of MVD is increasingly being recognized. The coronary microvasculature holds 90% of the total myocardial blood volume.^20^ MVD is implicated in angina with no obstructive coronary disease, also known as ‘syndrome X’ or microvascular angina, which can lead to ventricular dysfunction even in those with normal epicardial arteries. A recent study demonstrated evidence of coronary MVD in 68% of those attending the catheter laboratory with chest pain with no obstructive coronary disease and 39–53% of those with concomitant epicardial disease.^21^ MVD is of prognostic importance in acute myocardial infarction,^22^ myocardial infarction with no obstructive coronary artery disease,^23^ cardiomyopathy,^24^^,^^25^ cardiac transplantation,^26^ and heart failure with preserved ejection fraction.^27^ A recent randomized controlled trial demonstrated that coronary MVD responds well to stratified medical therapy.^9^ It is also hypothesized that coronary MVD may help to explain excess symptoms, risk and major adverse cardiac event in women,^28^ and the roughly 20% rate of persistent angina despite epicardial revascularization with PCI.^29–31^ Because routine invasive testing with angiography and pressure-derived FFR/iFR overlook the microvascular physiology, virtuQ may have a valuable role in providing the necessary additional parameters to better characterize coronary pathophysiology, improve diagnosis of MVD and better stratify treatment in these patients.

Because IHD results from a reduction in coronary blood flow, developing a method for measuring flow has been a scientific goal for many years. Until recently, this has meant using Doppler ultrasound or thermodilution but these indirect measures have proved impractical, technically challenging and inaccurate and have not been adopted into routine practice.^32–35^ The challenges of maintaining an optimum Doppler signal are well documented^36–38^ and the drawbacks widely acknowledged, even by those who advocate incorporating flow into physiological coronary assessment.^17^^,^^38–40^ Misalignment of the transducer may underestimate flow velocity. This was observed in the current study despite painstaking positioning *in vitro*. Doppler signal is sensitive to small movements and artefact is common.^36^^,^^39^ Whilst these errors may ‘cancel out’ in the calculation of CFR (ratio of two velocities), indices such as hyperaemic SR (HSR = P_d_/Doppler velocity) are far more susceptible to these errors. Kousera *et al.*^17^ used CFD simulation to predict the pressure-flow relationship in patients with coronary disease but underestimated pressure drop, likely because of error in the Doppler measurements, upon which their model was critically dependent. CFR is somewhat resistant to these errors if the magnitude of baseline and hyperaemic error remain unchanged. Indices such as hyperaemic or baseline SR ($\frac{P_{a}-P_{d}}{\text{APV}}$), hyperaemic myocardial resistance ($\frac{P_{d}}{\text{APV}}$) and index of myocardial resistance ($P_{d}\cdot \;\text{mean}\;\text{transit}\;\text{time}$) are far more susceptible to error in the Doppler- or thermodilution-derived flow estimation. Recently, an improved thermodilution method has been introduced that uses a monorail infusion catheter and a thermo- and pressure-sensitive wire.^41–43^ This method can measure absolute flow and MVR but requires dedicated hardware, reports hyperaemic flow at the catheter location and is associated with wider limits of agreement than the Q_CFD_ method (-37 to +24 vs. −4.7 to +8.8 mL/min), although the authors acknowledge that the virtuQ method is at an earlier stage of development and testing. We believe virtuQ to be the first non-Doppler and non-thermodilution invasive method for predicting coronary flow to be described.

Pressure-derived CFR is known to correlate closely with CFR derived from absolute flow as originally demonstrated in a canine model by Akasaka *et al*.^18^ In this study, CFR derived from Q_CFD_ correlated closely with pressure-derived CFR, suggesting Q_CFD_ was an accurate measure of absolute coronary flow. While the correlation was strong, CFR derived from Q_CFD_ was consistently greater than pressure-derived CFR. This is interesting and reassuring because the same observation was made by MacCarthy *et al*.^44^ in their experiment comparing thermo- and Doppler-derived CFR to pressure-derived CFR. The discrepancy is likely explained by the fact that the calculation of pressure-derived CFR neglects frictional energy losses, which our method fully captures.

Clinical data required for the virtuQ method are angiographic images and standard pressure-wire measurements, methods with which interventionists are already routinely familiar. Standard pressure wires tend to have better handling characteristics than those with combined Doppler or thermosensitive transducers. virtuQ requires no additional hardware, wires or infusions. A comprehensive physiological and anatomical assessment is generated (FFR, Q_CFD_, MVR, SR, and CFR) under baseline and hyperaemic conditions and this can be visualized in a user-friendly software environment. Whereas existing techniques estimate surrogate markers of flow (e.g. velocity or mean transit time) and incorporate these into ratios or indices, virtuQ determines flow and resistance in absolute units.

CFD modelling is increasingly being applied to cardiovascular medicine to characterize and predict human vascular pathophysiology which is poorly approximated by simpler fluid dynamic equations such as those of Bernoulli and Poiseuille.^14^^,^^44^^,^^45^ Perhaps the best example is virtual FFR (vFFR) computed from angiography. The accuracy of any CFD model is critically dependent upon tuning parameters that represent the physiological conditions of an individual patient, i.e. boundary conditions.^46–48^ When computing vFFR, the boundary conditions are unknown and assumptions have to be made. This limits accuracy. This is not a problem for virtuQ because the boundary conditions are known precisely in all cases. Thus, assumptions and therefore error are reduced.

### 4.1 Limitations

In the *in vitro* experiment, the 3D printed coronary models were rigid. The same is true of the computational model. However, we expect the overall effect coronary compliance to be negligible, especially in the context of a steady flow simulation and diseased vessels. Furthermore, previous CFD modelling work suggests a rigid assumption is acceptable in this context and does not adversely affect accuracy.^14^^,^^49–51^ At the current stage of development, the model does not account for flow to side branches which underestimates flow in more proximal segments. This is the opposite of the over-the-wire catheter infusion method because this predicts proximal but not distal flow. Future work will improve this by quantifying the flow lost to proximal branches. Because the simulation boundary conditions are known precisely, Q_CFD_ accuracy is dependent chiefly on the reconstruction protocol. In this study, we evaluated Doppler, as a clinically approved comparator, and found it lacking; a similar evaluation of thermodilution derived markers of flow would also be valuable.^42^ A potential limitation of the Q_CFD_ method as a clinical tool is the requirement for a pressure gradient of at least 4 mmHg in the epicardial artery to drive the CFD simulation. Theoretically, this means Q_CFD_ cannot be used in completely normal coronary arteries. Assuming a mean arterial pressure of 90 mmHg, Q_CFD_ will be accurate in cases where FFR is ≤0.95, i.e. the majority of cases studied in the catheter laboratory. This will affect baseline measurements more than hyperaemic. Ideally, coronary flow would be interpreted in light of the mass of myocardium subtended by that artery. However, there are currently no methods for measuring this in the cardiac catheter laboratory. Non-invasive techniques such as PET or CMR may have a role but are imprecise concerning the location of a stenosis.

## 5. Conclusions

Absolute coronary blood flow can be determined during standard angiography and pressure wire assessment. This novel method provides a comprehensive coronary physiological assessment of flow, pressure and resistance, across the entire coronary circulation, without the need for additional hardware, catheters, wires, or infusions. Using the novel method, epicardial and microvascular disease can be discriminated and quantified.

## Data availability

The data underlying this article are available in the article and in its online supplementary material.

## Supplementary material

Supplementary material is available at *Cardiovascular Research* online.

## Authors’ contributions

All authors made a substantial contribution either to the conception or design of the work (P.D.M., A.J.N., R.D.H., I.Z., P.V.L.), the acquisition and/or analysis of data (I.Z., H.E., L.A.-R., R.G., K.C., P.D.M.), interpretation of data (P.D.M., A.J.N., H.E., I.Z.), drafting the work (P.D.M., A.J.N., P.V.L., R.G.), or revising the work critically for important intellectual content (P.C.E.).

**Translational perspective**

Current pressure wire-based methods of assessing coronary disease cannot assess absolute flow or microvascular disease. Our novel absolute coronary flow (Q_CFD_) method, using only angiography-based computational fluid dynamics and a pressure wire, simultaneously measures fractional flow reserve, absolute coronary blood flow rate, microvascular resistance, and coronary flow reserve. Q_CFD_ is suitable for use in the catheter laboratory and requires no dedicated catheters, wires or infusions. Q_CFD_ measures blood flow and microvascular resistance in absolute units and allows microvascular and epicardial disease to be differentiated, quantified and separately assessed, with the potential to improve diagnostic accuracy and clinical management.

## Supplementary Material

cvaa220_Supplementary_Data
