# Supplementary material for: A novel method for measuring absolute coronary blood flow and microvascular resistance in patients with ischaemic heart disease
Source: Cardiovasc Res. 2020 Jul 14;117(6):1567–77. doi: 10.1093/cvr/cvaa220 (PMC8152717; doi:10.1093/cvr/cvaa220)
Supplement: cvaa220_Supplementary_Data [file cvaa220_supplementary_data.docx]

# **SUPPLEMENTAL MATERIAL**

# **A Novel Method for Measuring Absolute Coronary Flow Rate & Microvascular Resistance in Patients with Ischaemic Heart Disease.**

Morris PD *et al*

**Comprehensive Coronary Physiological Assessment**

**MS# CVR-2020-0350**

**3-D Printed Coronary Models**

Data were collected from five patients undergoing coronary angiography and FFR assessment. The patient’s diseased coronary arteries were reconstructed in 3-D. 3-D computer files were then used to 3-D print the patient’s diseased arteries by laser stereolithography in TuskXC2700T material with a layer thickness of 0.1mm (Materialise NV, BE). Cases and models are described below in the table.

| **Artery** | **Angiogram** | **3-D Printed artery** | **Details** |
| --- | --- | --- | --- |
| Case 1 LAD | 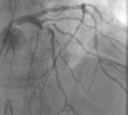 | 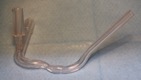 | FFR = 0.64  Inlet diameter = 4.78  Stenosis diameter = 1.36  Outlet diameter = 2.24  % stenosis = 71.6  Length = 68 mm |
| Case 2 RCA | 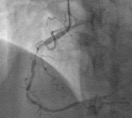 | 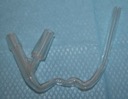 | FFR = 0.79  Inlet diameter = 4.44  Stenosis diameter = 1.28  Outlet diameter = 1.74  % stenosis = 71.1%  Length = 75 mm |
| Case 3 LAD | 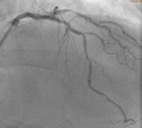 | 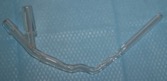 | FFR = 0.72  Inlet diameter = 3.54  Stenosis diameter = 1.18  Outlet diameter = 2.06  % stenosis = 66.8%  Length: 84 mm |
| Case 4 RCA | 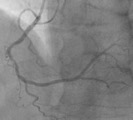 | 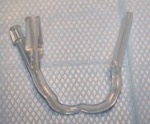 | FFR = 0.86  Inlet diameter = 3.16  Stenosis diameter = 1.70  Outlet diameter = 2.50  % stenosis = 46.2%  Length = 68 mm |
| Case 5 LCX | 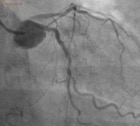 | 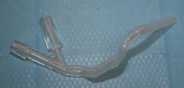 | FFR = 0.82  Inlet diameter = 3.13  Stenosis diameter = 1.66  Outlet diameter = 1.99  % stenosis = 47.1%  Length = 69 mm |

**Supplemental Table S1.** **Angiographic, physiological and geometric details of the 3-D printed coronary arterial models.** All measurements are in mm. LAD, left anterior descending artery; RCA, right coronary artery; LCX, left circumflex artery.

**Steady vs pulsatile in vitro analysis**

In the in vitro experiment pulsatility was imposed by the pulse modulator controlled by WinTest Controls software. The steady gear pump delivered the calibrated mean flow rate and the manifold imposed pulsatility via an internal bellows displacement method. The compliance chamber eliminated high frequency artefact. Using the WinTest controls software, pulsatility (Q) was controlled by prescribing the variation in volume (V) of the manifold as a function of time (t):

$\text{Q=}\text{ }\frac{\text{dV}}{\text{dt}}\text{= }\frac{\text{V}_{\text{t}}\text{-}\text{V}_{\text{t-1}}}{\text{dt}}$ and so $\text{V}_{\text{t}}\text{ = }\left( \text{Q}_{\text{t}} \text{- }\bar{\text{Q}} \right)\text{ ∙ dt + }\text{V}_{\text{t-1}}$

We applied a patient-specific modified sinusoidal flow waveform for each phantom thus.

$$\text{Q}_{\text{t}}\text{= }\bar{\text{Q}}\text{ + A ∙ sin (2π) ∙ }\frac{\text{t}}{\text{T}}$$

Below, is an example of a transient analysis from a patient case demonstrates a dynamic flow result through a diseased LCX artery under baseline (lower black line) and hyperaemic (upper grey line) conditions, over two cardiac cycles. Flow peaks during diastole (0–0.6 s and 1.0–1.6 s).


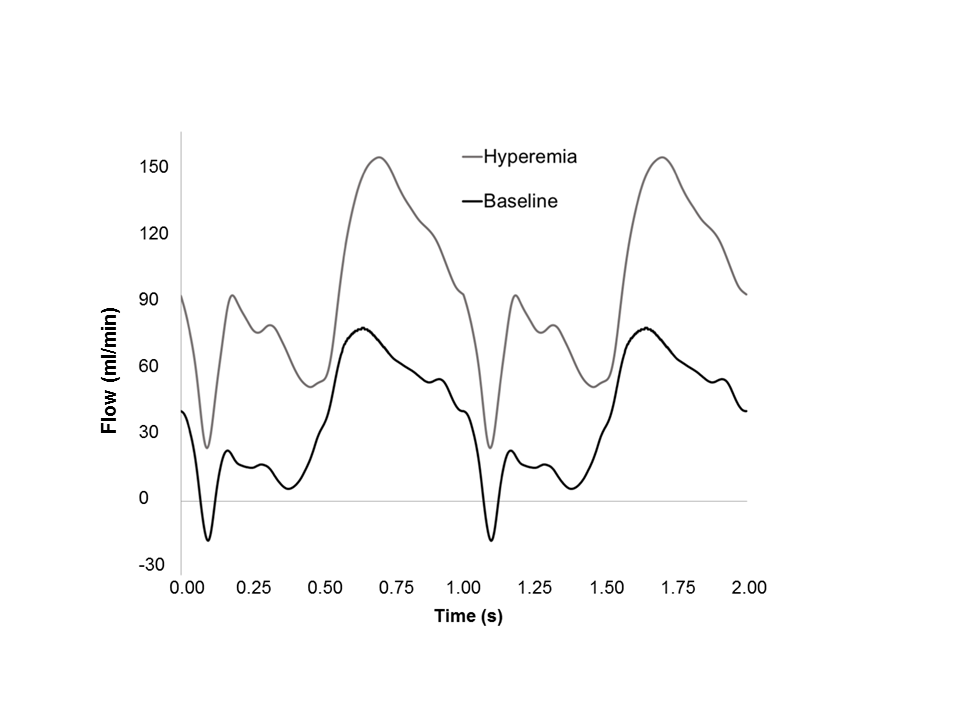


**Supplementary Figure S1.** Dynamic flow analysis derived from transient pressure boundary conditions.

**Table S2:** Figure 3a and figure 3b data (all values in mL/min)

| MODEL 1 | | MODEL 2 | | MODEL 3 | | MODEL 4 | | MODEL 5 | |
| --- | --- | --- | --- | --- | --- | --- | --- | --- | --- |
| MEAN | DIFFERENCE | MEAN | DIFFERENCE | MEAN | DIFFERENCE | MEAN | DIFFERENCE | MEAN | DIFFERENCE |
| 49.88 | -0.23 | 48.83 | -2.34 | 49.96 | -0.09 | 47.95 | -4.09 | 49.59 | -0.82 |
| 59.36 | -1.28 | 61.04 | 2.09 | 59.67 | -0.66 | 57.77 | -4.46 | 59.94 | -0.11 |
| 70.97 | 1.94 | 71.39 | 2.78 | 69.58 | -0.83 | 68.67 | -2.66 | 69.90 | -0.21 |
| 81.83 | 3.66 | 81.89 | 3.77 | 79.65 | -0.71 | 79.10 | -1.80 | 80.63 | 1.27 |
| 92.15 | 4.30 | 92.50 | 4.99 | 89.83 | -0.35 | 90.38 | 0.75 | 89.93 | -0.14 |
| 101.10 | 2.21 | 102.74 | 5.49 | 100.10 | 0.20 | 97.75 | -4.49 | 100.00 | 0.00 |
| 111.63 | 3.26 | 112.25 | 4.49 | 110.44 | 0.89 | 109.50 | -0.99 | 109.79 | -0.43 |
| 122.59 | 5.18 | 124.43 | 8.85 | 120.44 | 0.89 | 119.76 | -0.47 | 119.33 | -1.34 |
| 133.89 | 7.77 | 133.02 | 6.04 | 131.30 | 2.60 | 129.75 | -0.51 | 130.35 | 0.70 |
| 142.60 | 5.19 | 143.37 | 6.75 | 140.72 | 1.44 | 139.49 | -1.02 | 140.24 | 0.48 |
| 152.54 | 5.08 | 153.47 | 6.93 | 150.97 | 1.93 | 152.53 | 5.06 | 149.91 | -0.17 |
| 164.19 | 8.37 | 164.36 | 8.71 | 161.27 | 2.54 | 160.08 | 0.16 | 162.29 | 4.58 |
| 172.97 | 5.94 | 173.67 | 7.33 | 172.24 | 4.47 | 170.03 | 0.06 | 171.50 | 3.01 |
| 184.57 | 9.14 | 184.71 | 9.41 | 181.14 | 2.27 | 179.00 | -2.00 | 182.56 | 5.13 |
| Shaded cells denote values where Re > 500 | | | | | | | | | |

**Table S3.** Figure 3c and figure 3d data (all values in mL/min)

| MODEL 1 | | MODEL 2 | | MODEL 3 | | MODEL 4 | | MODEL 5 | |
| --- | --- | --- | --- | --- | --- | --- | --- | --- | --- |
| MEAN | DIFFERENCE | MEAN | DIFFERENCE | MEAN | DIFFERENCE | MEAN | DIFFERENCE | MEAN | DIFFERENCE |
| 42.96 | -14.14 | 55.20 | 10.36 | 48.63 | -2.78 | 54.03 | 8.01 | 44.63 | -10.78 |
| 52.45 | -15.17 | 65.63 | 11.19 | 57.57 | -4.91 | 65.70 | 11.34 | 52.71 | -14.63 |
| 60.14 | -19.79 | 76.05 | 12.02 | 66.51 | -7.04 | 73.05 | 6.04 | 61.95 | -16.17 |
| 67.84 | -24.41 | 85.70 | 11.31 | 77.91 | -4.25 | 81.19 | 2.31 | 69.64 | -20.79 |
| 75.53 | -29.03 | 95.34 | 10.59 | 89.31 | -1.46 | 90.12 | 0.15 | 79.26 | -21.56 |
| 85.02 | -30.06 | 103.44 | 6.78 | 99.24 | -1.63 | 99.04 | -2.02 | 87.34 | -25.41 |
| 93.61 | -32.89 | 112.32 | 4.52 | 107.68 | -4.74 | 107.18 | -5.75 | 95.42 | -29.26 |
| 102.20 | -35.72 | 119.64 | -0.84 | 117.12 | -5.89 | 116.11 | -7.91 | 103.89 | -32.34 |
| 109.90 | -40.34 | 129.29 | -1.56 | 126.06 | -8.02 | 125.42 | -9.29 | 117.74 | -24.65 |
| 118.49 | -43.17 | 138.93 | -2.27 | 133.03 | -14.09 | 132.78 | -14.59 | 125.82 | -28.50 |
| 127.08 | -45.99 | 148.58 | -2.99 | 141.47 | -17.21 | 143.27 | -13.61 | 134.67 | -30.81 |
| 135.67 | -48.82 | 159.00 | -2.15 | 148.44 | -23.27 | 150.62 | -18.91 | 143.52 | -33.12 |
| 143.36 | -53.44 | 169.43 | -1.32 | 156.40 | -27.37 | 158.37 | -23.43 | 151.60 | -36.97 |
| 152.85 | -54.48 | 179.07 | -2.04 | 164.35 | -31.47 | 167.69 | -24.81 | 160.83 | -38.51 |
| Shaded cells denote values where Re > 500 | | | | | | | | | |

**Table S4.** Figure 4 data. Flow rate (mL/min) vs pressure (mmHg) for each model.

| MODEL 1 | | | | MODEL 2 | | | | MODEL 3 | | | | MODEL 4 | | | | MODEL 5 | | | |
| --- | --- | --- | --- | --- | --- | --- | --- | --- | --- | --- | --- | --- | --- | --- | --- | --- | --- | --- | --- |
| Experimental | | CFD | | Experimental | | CFD | | Experimental | | CFD | | Experimental | | CFD | | Experimental | | CFD | |
| 50 | 3.7 | 49.8 | 3.7 | 50 | 9.1 | 47.7 | 9.0 | 50 | 8.7 | 49.9 | 8.7 | 50 | 3.7 | 45.9 | 3.7 | 50 | 4.7 | 49.2 | 4.7 |
| 60 | 4.7 | 58.7 | 4.7 | 60 | 12.6 | 62.1 | 12.7 | 60 | 11.0 | 59.3 | 11.0 | 60 | 4.7 | 55.5 | 4.7 | 60 | 6.0 | 59.9 | 6.0 |
| 70 | 6.3 | 71.9 | 6.3 | 70 | 15.7 | 72.8 | 15.7 | 70 | 13.7 | 69.2 | 13.7 | 70 | 6.0 | 67.3 | 6.0 | 70 | 7.3 | 69.8 | 7.3 |
| 80 | 8.0 | 83.7 | 8.0 | 80 | 19 | 83.8 | 19.0 | 80 | 16.7 | 79.3 | 16.7 | 80 | 7.3 | 78.2 | 7.3 | 80 | 9.0 | 81.3 | 9.0 |
| 90 | 9.7 | 94.3 | 9.7 | 90 | 23 | 95.0 | 22.7 | 90 | 20.0 | 89.7 | 20.0 | 90 | 9.0 | 90.8 | 9.0 | 90 | 10.3 | 89.9 | 10.3 |
| 100 | 11.0 | 102.2 | 11.0 | 100 | 26.3 | 105.5 | 26.3 | 100 | 23.7 | 100.2 | 23.7 | 100 | 9.7 | 95.5 | 9.7 | 100 | 12.0 | 100.0 | 12.0 |
| 110 | 13.0 | 113.3 | 13.0 | 110 | 30.3 | 114.5 | 29.7 | 110 | 27.7 | 110.9 | 27.7 | 110 | 11.7 | 109.0 | 11.7 | 110 | 13.7 | 109.6 | 13.7 |
| 120 | 15.3 | 125.2 | 15.3 | 120 | 34.5 | 128.9 | 35.3 | 120 | 31.7 | 120.9 | 31.7 | 120 | 13.3 | 119.5 | 13.3 | 120 | 15.3 | 118.7 | 15.3 |
| 130 | 18.0 | 137.8 | 18.0 | 130 | 38.9 | 136.0 | 38.3 | 130 | 36.7 | 132.6 | 36.7 | 130 | 15.0 | 129.5 | 15.0 | 130 | 17.7 | 130.7 | 17.7 |
| 140 | 20.0 | 145.2 | 19.7 | 140 | 43.4 | 146.7 | 43.0 | 140 | 40.7 | 141.4 | 40.7 | 140 | 16.7 | 139.0 | 16.7 | 140 | 19.7 | 140.5 | 19.7 |
| 150 | 22.0 | 155.1 | 22.0 | 150 | 48.1 | 156.9 | 47.7 | 150 | 45.7 | 151.9 | 45.7 | 150 | 19.7 | 155.1 | 19.7 | 150 | 21.7 | 149.8 | 21.7 |
| 160 | 25.3 | 168.4 | 25.3 | 160 | 53.1 | 168.7 | 53.3 | 160 | 51.0 | 162.5 | 51.0 | 160 | 20.7 | 160.2 | 20.7 | 160 | 25.0 | 164.6 | 25.0 |
| 170 | 27.0 | 175.9 | 27.3 | 170 | 58.1 | 177.3 | 57.7 | 170 | 57.3 | 174.5 | 57.3 | 170 | 22.7 | 170.1 | 22.7 | 170 | 27.0 | 173.0 | 27.0 |
| 180 | 31.0 | 189.1 | 31.0 | 180 | 63.3 | 189.4 | 64.0 | 180 | 61.7 | 182.3 | 61.7 | 180 | 24.3 | 178.0 | 24.3 | 180 | 30.0 | 185.1 | 30.0 |
